# Thyroid Hormone Action in Muscle Atrophy

**DOI:** 10.3390/metabo11110730

**Published:** 2021-10-25

**Authors:** Maria Angela De Stefano, Raffaele Ambrosio, Tommaso Porcelli, Gianfranco Orlandino, Domenico Salvatore, Cristina Luongo

**Affiliations:** 1 Department of Clinical Medicine and Surgery, University of Naples Federico II, 80131 Naples, Italy; m.angeladestefano@gmail.com; 2 Istituti di Ricovero e Cura a Carattere Scientifico, SDN, 80143 Naples, Italy; ambrosioraf.ra@gmail.com; 3 Department of Public Health, University of Naples Federico II, 80131 Naples, Italy; tommasoporcelli@gmail.com; 4 Azienda Ospedaliera Universitaria Federico II, 80131 Naples, Italy; gianfranco_orlandino@hotmail.com

**Keywords:** thyroid hormone, deiodinase, muscle atrophy

## Abstract

Skeletal muscle atrophy is a condition associated with various physiological and pathophysiological conditions, such as denervation, cachexia, and fasting. It is characterized by an altered protein turnover in which the rate of protein degradation exceeds the rate of protein synthesis, leading to substantial muscle mass loss and weakness. Muscle protein breakdown reflects the activation of multiple proteolytic mechanisms, including lysosomal degradation, apoptosis, and ubiquitin–proteasome. Thyroid hormone (TH) plays a key role in these conditions. Indeed, skeletal muscle is among the principal TH target tissue, where TH regulates proliferation, metabolism, differentiation, homeostasis, and growth. In physiological conditions, TH stimulates both protein synthesis and degradation, and an alteration in TH levels is often responsible for a specific myopathy. Intracellular TH concentrations are modulated in skeletal muscle by a family of enzymes named deiodinases; in particular, in muscle, deiodinases type 2 (D2) and type 3 (D3) are both present. D2 activates the prohormone T4 into the active form triiodothyronine (T3), whereas D3 inactivates both T4 and T3 by the removal of an inner ring iodine. Here we will review the present knowledge of TH action in skeletal muscle atrophy, in particular, on the molecular mechanisms presiding over the control of intracellular T3 concentration in wasting muscle conditions. Finally, we will discuss the possibility of exploiting the modulation of deiodinases as a possible therapeutic approach to treat muscle atrophy.

## 1. Introduction

Thyroid hormone (TH) affects virtually every organ system in the body, including skeletal muscle. Among its widespread biological functions, TH controls tissue development and homeostasis, cellular growth, differentiation, and metabolism [1]. In particular, specific TH levels are critical for the development of different tissues and are essential for the regulation of metabolic processes in life.

Skeletal muscle is one of the largest tissues in humans, accounting for about 40% of body weight. It is a dynamic and plastic tissue that is primarily responsible for locomotion, but it also plays important roles in many other physiological processes such as glucose and energy metabolism. The skeletal muscle is a well-known TH target tissue; TH affects muscle development, contractile function, myogenesis, and bioenergetic metabolism [2]. Alterations of circulating TH levels are associated with several muscle symptoms and signs. Since the 1970s, many studies have shown that thyroid disorders could induce a specific muscle myopathy. Notably, THs have been demonstrated to be one of the elements controlling muscle mass, and their alteration could be responsible for the development of muscle atrophy [3,4,5]. 

Muscle atrophy is a common comorbidity factor among patients with chronic and/or advanced disease but can also be induced by muscle disuse or muscle dystrophies. The resulting condition derives from the combined effects of muscle atrophy and muscle stem cell death, which lead to an overall loss of muscle mass and a decrease in muscle strength [6]. In catabolic conditions, protein breakdown is enhanced and exceeds protein synthesis, thereby resulting in myofiber atrophy [7]. THs control many processes involved in the development of muscle atrophy. Here, we will review the present knowledge of the role of TH in skeletal muscle atrophy. In particular, we will discuss the molecular mechanisms presiding over the control of local intracellular T3 concentration in muscle and how these are involved in the development of muscle atrophy.

### 1.1. TH Action

The thyroid gland secretes mainly thyroxine (T4) and, in small amounts, triiodothyronine (T3) [1]. T3 is the most metabolically active form of TH, whereas T4, due to its low affinity for TH receptors, is considered a prohormone and constitutes a large reserve of T3. It was long unclear how a pleiotropic agent such as TH could regulate a multitude of cellular processes in virtually all cells of the body without grossly modifying their circulating levels. Indeed, homeostatic regulation, operated by the hypothalamic–pituitary–thyroid axis (HPT axis), guarantees a very stable concentration of circulating TH under healthy conditions [8,9]. When circulating TH levels are too high or too low, the hypothalamus and pituitary gland can modulate thyroid-stimulating hormone (TSH) and the TSH-releasing hormone secretion to compensate for this unbalance through the negative feedback exerted by TH. Beyond this central regulation of circulating TH levels, there is a second system that acts at the intracellular level, modulating the amount of intracellular TH. Different cells, even if exposed to the same amount of circulating TH, can have a dramatically different thyroid status from each other, and that condition can be rapidly modified. The discovery of TH transporters (THT) and deiodinases, together with a better understanding of the peripheral metabolism of TH, dramatically changed our understanding of TH action [1,10]. The TH intracellular concentration depends on the expression of THT and deiodinases. 

Three different families of THT have been identified: monocarboxylate transporter (MCT), organic anion-transporting polypeptide (OATP), and L-type amino acid transporters (LAT). THT can differ for TH affinity and tissue distribution. MCT8, the first THT identified, equally transports T4 and T3, and is expressed in the heart, skeletal muscle, kidney, liver, placenta, and testis, whereas OATP1C1 is a high-affinity transporter for T4 and is mainly expressed in the brain. The physiological relevance of THTs has been proven by evidence that mutations in humans in MCT8 and MCT10 genes (decreasing their activities) are associated with psychomotor retardation (Allan–Herndon–Dudley syndrome) and neurodegenerative diseases, respectively [10,11]. 

Once inside the cell, the intracellular concentration of TH is tightly controlled by deiodinases. Deiodinases type 1 and type 2 (D1 and D2) are able to activate T4 into T3. Deiodinase type 3 (D3) is the principal physiological inactivator of TH; it prevents the activation of T4 by converting it into reverse-T3 (rT3) or terminates T3 action by converting it into T2. D1 is expressed in the thyroid, liver, and kidney, and it is the only deiodinase capable of both activating and inactivating TH. D1 is induced by TH. D2 is expressed in key thyroid-responsive tissues, such as brain tissue, brown adipose tissue, and, relevant to this review, in skeletal muscle, wherein D2 can rapidly enhance TH signaling by increasing the intracellular production of T3. D2 has a very short half-life (about 30 min); moreover, T4 increases the proteasome-mediated degradation of D2, while T3 decreases its expression, thus making D2 a very sensitive rheostat to thyroid status. D3 is mainly expressed in fetal tissues; its expression declines after birth, and in adult life, it is restricted to brain, placenta, skin, and pancreatic b-cells. However, D3 expression can be reactivated in different tissues during specific physiological processes, such as muscle regeneration, or by pathological conditions, such as tumors. Taken together, the actions of the three deiodinases constitute a potent mechanism for pre-receptor regulation of TH action at the cellular level [1]).

Intracellular T3 exerts its biological activity by binding to TH nuclear receptors (TRs) and regulating target gene expression (genomic action), but also by binding to cytosolic proteins and activating intracellular signals (non-genomic action). TRs function as ligand-modulated transcription factors. The binding of T3 to TRs modifies the transcription of target genes by an exchange between co-repressor and coactivator complexes. TRs are encoded by two genes, Thra and Thrb [12]. They are differentially expressed by tissues and during development. TRa is mainly expressed in skeletal muscle, heart, intestine, brain, and skeleton tissue, whereas TRb is mainly expressed in pituitary and liver tissue.

During the last ten years, it has been demonstrated that the specific modulation of local TH concentration is essential to the progression of complex processes, such as thermogenesis, myogenesis, and neurogenesis [13,14,15,16,17]. D2 and D3 are both expressed in muscle tissue as well as in thyroid hormone transporters MCT8 and MCT10 and thyroid hormone receptor TRα1. The generation of genetically modified mouse models has shed light on the role of the local control of TH concentration as a key regulator of muscle development, metabolism, and homeostasis (Table 1).

### 1.2. Effects of TH on Skeletal Muscle Physiology

**Figure 1:**
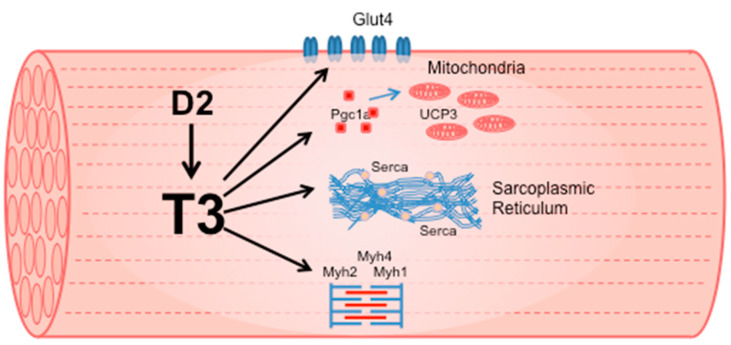
TH induces the expression of multiple genes that code for proteins essential to defining muscle contractile and metabolic properties.

TH induces the expression of multiple genes that code for proteins essential to defining muscle contractile and metabolic properties (Figure 1). The molecular effects of TH on skeletal muscle are evident from development, when TH and neuronal innervation trigger the transition of muscle fibers from the embryonic/neonatal phenotype to the adult phenotype. Notably, TH stimulates the conversion of muscle fiber type from slow to fast by inducing the transition of MYH7 to MYH2, MYH2 to MYH1, and MYH1 to MYH4 [22]. Hypothyroid conditions delay this switch, as the only Thra deletion in mice is sufficient to increase MYH7 expression [20]. However, slow muscle is considered more sensitive to TH alterations than fast muscle. Rat slow muscle, such as soleus, has higher T3 levels compared to fast extensor digitorum longus muscle (EDL), even if T4 levels are similar [23]. This is in agreement with the higher Dio2 expression in slow-twitch than fast-twitch mouse skeletal muscle [24]. Nonetheless, in a mouse model of Dio2 global knockout (D2KO), unexpectedly, the absence of Dio2 in soleus muscle induced a marked increase in the number of fast, glycolytic type IIB fibers [18]. Fast fibers are more glycolytic, whereas slow fibers are oxidative and have more mitochondria. Interestingly, peroxisome proliferator-activated receptor coactivator 1α (PGC-1a) gene expression, a TH-dependent regulator of slow muscle fiber type, was decreased by 50% in D2KO soleus [18] compared to WT. PGC1a is involved in mitochondrial biogenesis and favors oxidative metabolism by increasing mitochondrial biogenesis [25]. Thus, the reduction in PGC1a expression in D2KO slow-twitch muscle could explain the increase in fast glycolytic fibers. However, the single overexpression of Dio2 in the C2C12 muscle cell line induces a shift from oxidative phosphorylation to glycolysis [26]. 

TH also plays an important role in myogenesis, a multistep process responsible for normal skeletal muscle development, maintenance, and repair of adult myofibers. The initial step consists of the activation of satellite cells, their amplification, differentiation, and fusion to form new myofibers. The correct progression through these phases is orchestrated by myogenic regulatory factors (MRFs) such as Myf5, MyoD, and Myogenin. Several studies showed that, more than the plasmatic T3 levels, a finely tuned sequential expression of D3 and D2 is important than plasmatic T3 levels to customizing the intracellular amount of TH in muscle stem cells (satellite cells) during the different phases of myogenic lineage. Satellite cells and C2C12 cells cultured under proliferative conditions express elevated D3 levels that decline upon differentiation [13,14]. D3 genetic ablation from satellite cells increases intracellular T3 concentration and induces massive apoptosis through the activation of the FoxO3 signaling pathway [13,14]. D2 has an opposite pattern of expression versus D3. Dio2 expression increases during differentiation, similar to MyoD. The genetic ablation of Dio2 in these cells impairs myogenic differentiation, a phenotype that is reverted by culturing the cells in the presence of T3 [13]. 

These studies highlight the complex role of local control of TH in muscle physiology. Of note, it is evident from these studies that the plasmatic concentration of the active hormone T3 is not adequate for the different processes in which stem cells are involved. Accomplishing these functions requires customizing T3 concentrations, which must be dynamically regulated by the specific action of deiodinases as well as the expression of TH transporters and receptors.

### 1.3. Muscle Atrophy and Thyroid Dysfunction

Muscle atrophy is a condition characterized by a decrease in muscle mass. Different causes can be responsible for muscle atrophy, ranging from progressive muscle disuse associated with aging, muscle diseases such as dystrophies, or systemic diseases such as cancer, sepsis, or burn injury. The loss in muscle mass is associated with a loss in strength, muscle weakness, and hypofunction, which make the subject more fragile [27]. The balance between protein synthesis and degradation, and the ability to regenerate muscle fibers, are some of the elements that determine muscle mass. Several factors affect this balance, e.g., physical activity, nutritional status, hormones, and diseases. The impact of TH on muscle physiology is shown by the muscular consequences of systemic hyperthyroidism or hypothyroidism, as well as by mutations impairing the function of even just one of the genes involved in intracellular TH signaling (i.e., receptors or transporters) [13,28]. 

Hypothyroidism and hyperthyroidism are conditions associated with a spectrum of muscle symptoms and signs, ranging from myalgias, cramps, and easy fatigability to atrophic myopathy and rhabdomyolysis. The severity of the myopathy correlates with the severity and the duration of the hypothyroidism or hyperthyroidism [4,5]. 

Hypothyroid subjects show a characteristic myopathy especially affecting type II fibers. The most severely clinically affected patients show type II muscle fiber atrophy and loss, with increased central nuclear counts and the presence of mitochondrial abnormalities [29]. Overt and subclinical hyperthyroidism is associated with a decrease in muscle strength and cross-sectional area that usually improves after treatment with anti-thyroid drugs. It is thought that muscle atrophy observed in hyperthyroid patients depends on a faster protein turnover. The hyperthyroid state increases basal metabolism and, therefore, energy expenditure. Thus, the extra energy demands are satisfied by the augmented oxidation of lipids and proteins. The catabolic effect on protein metabolism causes accelerated protein breakdown and thus atrophy. The evidence that mild degrees of thyroid dysfunction might be associated with muscle alterations suggests that this condition should probably be treated, especially in a fragile population such as the elderly. Sarcopenia is an age-related muscle-atrophic condition that is due to a reduction in muscle regeneration and a progressive decrease in anabolism with an increase in catabolism. The prevalence of subclinical thyroid disorders increases with age. Interestingly, subclinical hyperthyroidism, but not subclinical hypothyroidism, has influences on muscle mass and strength in elderly subjects. In an elderly Korean population, it has been observed that higher levels of T4 are associated with sarcopenia. A Chinese study also showed that higher free triiodothyronine (FT3) concentrations within a normal range are correlated to muscle mass and muscle function in elderly subjects [30]. However, low FT3 has been identified as a possible marker of frailty in the elderly [31]. It has been proposed that TH deficiency might act at different levels (nerve, neuro-muscular junction, and muscle fiber) [32] through the alteration of glycogenolytic and oxidative metabolism, expression of contractile proteins, and neuro-mediated damage. 

It is interesting to observe that not only systemic alterations in TH concentration but also mutations of single genes coding for some of the proteins involved in TH signaling are associated with muscle disorders. Thyroid hormone transporter MCT8 deficiency is an inherited disorder that is characterized by severe intellectual disability, an impaired ability to speak, diminished muscle tone (hypotonia), muscle wasting, and/or movement abnormalities. SECIS-binding protein 2 (SBP-2) is involved in the synthesis of selenoproteins as deiodinases. Deficits of SBP2 are associated with thyroid dysfunction and neurocognitive deficits, as well as azoospermia, muscular dystrophy, photosensitivity, and immune dysfunction [33]. 

Understanding the molecular mechanisms through which TH controls muscle mass will allow a better comprehension of the development of muscle atrophy and will eventually allow the identification of therapeutic targets for the treatment of muscle loss.

### 1.4. Effects of TH on Skeletal Muscle Pathophysiology

Skeletal muscle has a remarkable regenerative capacity and can undergo several rounds of repair in response to injury and/or pathophysiologic conditions such as sarcopenia or dystrophies [34,35]. Muscle regeneration is a process highly sensitive to TH concentration. In D2KO mice, muscle regeneration, induced by cardiotoxin-injury, is impaired despite the normal concentration of circulating TH, supporting the concept that increased requirements in TH at regeneration sites are satisfied by the local action of D2. Moreover, in D2KO mice, MyoD levels (a well-known TH responsive gene) are reduced, leading to a marked delay in muscle differentiation/regeneration. Thus, during regeneration, adequate MyoD levels are ensured by the local rise of TH mediated by D2. In skeletal muscle, D2 is positively regulated by FoxO3a, a forkhead transcriptional factor that is implicated in muscle differentiation and in the activation of autophagy. Accordingly, the expression of Dio2 is significantly reduced in FoxO3-null mice. Muscle stem cells from FoxO3-null mice fail to fully differentiate due to the absence of MyoD, and treatment with TH is able to rescue a complete myogenic differentiation. 

D3 is highly expressed in myoblasts during the initial phase of the regenerative process. D3 action reduces intracellular TH signaling, allowing the proliferation of satellite cells. Satellite cell-specific genetic D3 depletion severely compromises skeletal muscle regeneration, with irreversible stem cell death due to excessive intracellular TH levels. D3 plays a crucial role in the early phase of muscle regeneration, when cells have to be protected from the “normal” circulating TH that is sufficient to trigger a death pathway via the FoxO3–MyoD axis in these cells [14]. 

Muscle atrophy is the result of altered protein and cell turnover. Indeed, an increase in muscle turnover that leads to premature exhaustion of satellite cells or a reduction in regenerative potential due to a decrease in the satellite cell pool could be a cause of muscle atrophy. The number of satellite cells is progressively reduced during aging, impairing regenerative capacity and leading to the loss of muscle mass. Sarcopenia has been correlated with alteration in TH signaling. It has been observed that serum TH levels decrease with aging, and this seems to influence skeletal muscle physiology [36]. During aging, the reduction of muscle mass is associated with an overall shift from fast to slower fibers, a decrease in type 2b fibers, an increase of type 2x, and the conversion of type 2a to type 1 fibers [37,38,39]. These changes are similar to what is observed in hypothyroid conditions [40] and are consistent with the lower level of TH detected during aging. Interestingly, administration of T3 to aged rats induces an increase of Myh2 and Myh1 expression in the slow-twitch soleus, whereas no significant changes are observed in the fast-twitch EDL after the treatment [41]. TH controls mitochondrial function in skeletal muscle, which is also altered during aging [3]. Indeed, in muscle fibers, ATP production and mtDNA are reduced with aging [42,43,44]. In model muscle mice, p43 overexpression (the mitochondrial THRA isoform) stimulated mitochondrial biogenesis and activity, leading to increased oxidative stress and reduced mitochondrial function. This condition is probably the cause of muscle sarcopenia observed starting from 6 months of age [45].

In several muscular dystrophies, regenerative muscle processes are impaired, and this leads to muscle wasting, progressive weakness, and metabolic disorder. Duchenne muscular dystrophy (DMD) is the most common form of muscular dystrophy, caused by the absence of functional dystrophin. The dystrophic myofibers undergo continuous cycles of degeneration and regeneration, resulting in the loss of muscle tissue, a process likely due to a progressive decrease in the satellite cell reservoir. Intriguingly, alterations in TH circulating levels have a profound impact on the phenotype of a mouse model of Duchenne named mdx [46]. McIntosh and Anderson demonstrated that systemic hypothyroidism induces an increase in myogenic precursor cell proliferation but a delay in myotube formation, worsening the phenotype of mdx mice. Of note, McArdle and colleagues demonstrated that hypothyroidism induced by PTU (an anti-thyroid drug that significantly reduces plasma TH levels) improves the dystrophic phenotype, preventing necrosis in the muscle of 21-day old mdx mice and eliminating the characteristic elevation in serum creatine kinase (a marker of muscle damage). This study provided the first demonstration that experimental manipulation of TH levels alters the onset of necrosis in mdx mice [47]. It was later demonstrated that thyroid antagonist (PTU or MMI) treatments reduce the rate of muscle degeneration in a model of avian muscular dystrophy. Indeed, if hypothyroidism is established immediately after hatching, the muscle function of dystrophic chicks significantly improves. The beneficial effects of anti-thyroid drugs on avian dystrophy are specifically due to induced hypothyroidism since TH deprivation increases muscle function (righting ability) and reduces plasma CK activity in dystrophic chickens [48]. In contrast, in regenerating mdx muscle, hyperthyroidism induces an increase of necrosis and an early differentiation of myogenic precursor cells that impairs the growth and formation of new myotubes [49,50]. It is not known if this is the result of a decreased proliferation rate of the myogenic precursor cells or an increased and anticipated fusion of the cells. Taken together, these data are consistent with the role of intracellular TH signaling during myogenesis and indicate that changes in TH levels deeply impact the dystrophic phenotype. 

### 1.5. Common Pathways and Shared Molecular Mechanisms between TH and Muscle Wasting

**Figure 2:**
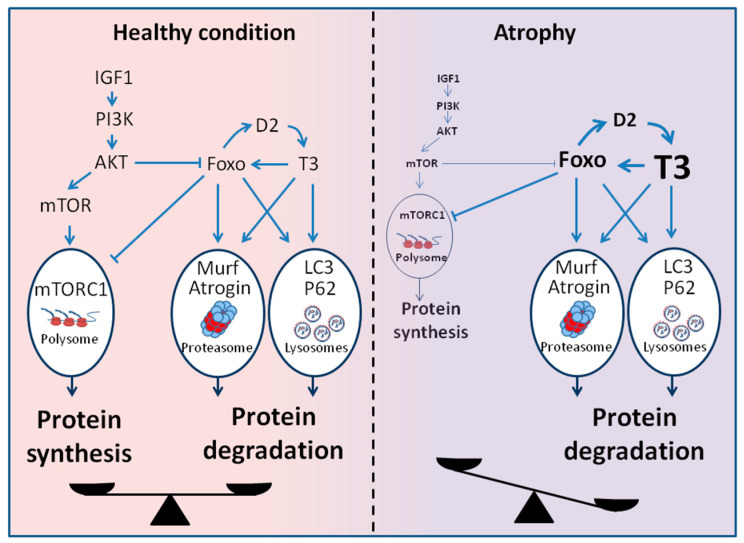
Signaling pathway involved in the regulation of the muscle mass. In healthy conditions, the balance between catabolic and anabolic signaling pathways allows the maintenance of muscle mass. In atrophic conditions, the rise in intracellular TH levels shifts this balance towards increased protein catabolism through the activation of canonical atrophic signaling pathways. (IGF1: insulin-like growth factor-1, PI3K: phosphatidylInositol 3-Kinase, AKT: protein kinase B, mTOR: mammalian target of rapamycin, mTORC1: mTOR Complex 1, LC3: microtubule-associated protein 1A/1B-light chain 3, P62: sequestosome 1, FoxO: forkhead transcription factors of the O class).

Although the etiologies of muscle wasting can be very different, a common feature is the activation of multiple proteolytic mechanisms, including lysosomal degradation, apoptosis, and ubiquitin–proteasome protein breakdown. In pathological conditions characterized by a catabolic state as cancer, heart failure, and sepsis, the increased energy requirements are satisfied by increased protein degradation, with consequent progressive muscle wasting. In particular, the ubiquitin-proteasome system, the autophagy–lysosome system, and Akt/FoxO signaling pathways have been intensively studied and implicated in these conditions. Under physiological conditions, TH stimulates both protein synthesis and degradation, whereas supraphysiological TH levels shift this balance towards increased protein catabolism. Interestingly, many connections between TH action and proteolytic pathways have been identified (Figure 2). 

The ubiquitin–proteasome system is one of the most important proteolytic systems controlling protein turnover in muscle. In atrophic muscle, an increased expression of ubiquitin-conjugating enzymes (E2), ubiquitin-protein ligase (E3), and proteasome subunits have been observed. Atrogin-1 and MuRF1 are two muscle-specific ubiquitin ligases whose expression is strongly increased in several catabolic muscle conditions and are considered master genes of muscle atrophy. Tawa et al. demonstrated a clear effect of TH in activating the proteasome-dependent proteolytic pathway during atrophy [51]. They showed that, in rats, hypothyroidism leads to a decrease in proteasome-dependent muscle protein degradation [51]. O’Neal et al. later demonstrated that T3 administration in rats upregulates the expression of atrogin-1 and MuRF1 in skeletal muscle [52]. It is not known whether the increase in protein degradation and the expression of atrogin-1 and MuRF1 is due to a direct or indirect effect of T3 [53,54]. However, overexpression of the mitochondrial TH receptor p43 in skeletal muscle induces atrophy and a strong increase in Atrogin-1 and MuRF1 expression [45].

Accumulating evidence has demonstrated that TH also induces the autophagy–lysosomal system in different tissues, such as liver and muscle. In 1978, De Martino and Goldberg were the first to associate the effects of TH on protein degradation with lysosomal enzyme activities in rat liver and skeletal muscle [55]. They observed that TH regulates protein degradation by increasing lysosomal enzyme activity, i.e., cathepsin B and D. In 2009, O’Neal et al. showed that this increase was responsible for the muscle wasting observed in hyperthyroidism [52]. Furthermore, TH increases autophagy in skeletal muscle by inducing the expression of key autophagy genes, including microtubule-associated protein light chain 3 (LC3), sequestosome 1 (p62), Unc-51-like kinase 1 (Ulk1), and FoxO1/3a [28]. Indeed, the expression of dominant-negative TRα mutant in skeletal muscle reduced autophagy, mitochondrial turnover, and altered muscle fiber phenotype [56].

It has been shown that the simultaneous activation and coordinate regulation of lysosomal and proteasomal pathways for protein degradation are mediated by Forkhead box O (FoxO) [57]. Sandri et al. were the first to implicate FoxO in the control of muscle size during atrophy [58,59]. In physiological conditions, the transcription factors FoxO are negatively regulated by the PI3K–AKT signaling pathway, which promotes protein synthesis. In muscle atrophy, the decreased activity of the PI3K/AKT pathway leads to the activation of FoxO. In particular, a specific transcription factor, FoxO3, induces transcription of the Atrogin-1 and a dramatic decrease in fiber size in different models of atrophy. Constitutively active FoxO3 induces the expression of atrogin-1 and the aberrant atrophy of myotubes and muscle fibers [58]. Conversely, when FoxO3 activity is blocked by a dominant-negative construct in myotubes or by RNAi in mouse muscles in vivo, atrogin-1 induction during starvation or glucocorticoid administration is hampered. We also demonstrated that FoxO3 is transcriptionally induced by TH, but as mentioned before, FoxO3 also indirectly sustains T3 concentration by inducing D2 [14].

Overall, these data suggest an interplay between TH muscle status and the development of muscle atrophy. 

## 2. Conclusions

Overall, TH has a large influence on muscle physiology, and it is clear that TH acts on different molecular pathways in a spatially and temporally regulated manner. The evidence that both hypothyroidism and hyperthyroidism alter muscle physiology, as well as recent data showing the relevance of intracellular control of TH action, implies that muscle TH concentration should be maintained in a narrow but dynamically regulated range. TH serves as a crucial regulator of the atrophic process by activating the canonical atrophic molecular pathways and controlling fiber regeneration. The local control of TH muscle concentration throughout deiodinase action makes it reasonable to exploit the modulation of deiodinases as a possible therapeutic approach to treat muscle-wasting disorders. Furthermore, the development of specific TH agonists represents a further scenario of intervention to modulate TH action at specific sites.

In the future, the development of selective deiodinase modulators will allow the modification of the intracellular concentration of TH, avoiding the systemic alteration of TH and thus the related side effects. One challenging aspect of the development of these modulators is the evidence that muscle fibers and cells could have different needs in TH during different phases of the same process. To this aim, it will be important to have very manageable molecules (i.e., with short half-lives, low IC_50_).

## Figures and Tables

**Table 1:** Muscular phenotype of murine thyroid-hormone-signaling knockout models.

Knockout	Muscular Phenotype	Reference
Dio2	Changes in contractile function and fiber type composition. In contrast with local hypothyroidism, mice show increased fast characteristics of soleus and increased myofiber CSA, contraction rate, and fatigue resistance.	[18]
Dio3 (Global KO)	Neonatal thyrotoxicosis followed by central hypothyroidism, decrease in body weight, and partial perinatal lethality.	[19]
Dio3 (MuSC specific KO)	Massive apoptosis of MuSC, impairment of the initial phases of muscle regeneration, and delay of repair process.	[14]
Thra/Thrb, Thra, Thrb	Similar to the absence of thyroid hormone, fast to slow MHC isoform switching and decrease in body and muscle weights.	[20]
Slc16a2 (Mct8)/ OATP1C1	Hyperthyroid state of skeletal muscle, shift from slow to fast fibers, muscle hypoplasia, and impaired muscle regeneration.	[21]
Slc16a2 (Mct8)	Hyperthyroid state of skeletal muscle, shift from slow to fast fibers, and impaired muscle regeneration.	[21]
Slc16a10 (Mct10)	Increased plasma and muscle and kidney aromatic amino acid.	[21]
